# Males are more sensitive to reward and less sensitive to loss than females among people with internet gaming disorder: fMRI evidence from a card-guessing task

**DOI:** 10.1186/s12888-020-02771-1

**Published:** 2020-07-07

**Authors:** Jialin Zhang, Yan Hu, Ziliang Wang, Min Wang, Guang-Heng Dong

**Affiliations:** 1 Center for Cognition and Brain Disorders, Institute of Psychological Research, Hangzhou Normal University, Hangzhou, 311121 Zhejiang Province China; 2 Department of Creative Technologies, Blekinge Institute of Technology, SE-371 79 Karlskrona, Sweden; 3 State Key Laboratory of Cognitive Neuroscience and Learning and IDG/McGovern Institute for Brain Research, Beijing, China; 4 Zhejiang Key Laboratory for Research in Assessment of Cognitive Impairments, Hangzhou, Zhejiang Province China

**Keywords:** Internet gaming disorder, Gender, Reward processing, Loss processing

## Abstract

**Background:**

Many studies have found an interesting issue in the Internet gaming disorder (IGD): males are always observed to be the majority. However, there are little research to exploring the differences in the neural mechanisms between males and females in decision-making process among people with IGD. Therefore, explore the reward/loss processing between different gender with IGD could help in understanding the underlying neural mechanism of IGD.

**Methods:**

Data from functional magnetic resonance imaging (fMRI) were collected from 111 subjects (IGD: 29 males, 25 females; recreational internet game user (RGU): 36 males, 21 females) while they were performing a card-guessing task. We collected and compared their brain features when facing the win and loss conditions in different groups.

**Results:**

For winning conditions, IGD group showed hypoactivity in the lingual gyrus than RGU group, male players showed hyperactivity in the left caudate nucleus, bilateral cingulate gyrus, right middle frontal gyrus (MFG), right precuneus and inferior parietal lobule relative to the females. And significant sex-by-group interactions results showed higher brain activities in the thalamus, parahippocampal gyrus and lower brain activities in Inferior frontal gyrus (IFG) were observed in males with IGD than females. For losing conditions, IGD group showed hypoactivity in the left lingual gyrus, parahippocampal gyrus and right anterior cingulate cortex (ACC) compared to the RGU group, male players showed hyperactive left caudate nucleus and hypoactive right middle occipital gyrus relative to females. And significant sex-by-group interactions results showed that compared to females with IGD, males with IGD showed decreased brain activities in the IFG and lingual gyrus.

**Conclusions:**

First, there appeared to be no difference in reward processing between the IGD and RGU group, but IGD showed less sensitivity to loss. Secondly, male players showed more sensitivity to rewards and less sensitivity to losses. Last but not least, males and females showed opposite activation patterns in IGD degree and rewards/losses processing. And male IGD subjects are more sensitive to reward and less sensitive to loss than females, which might be the reason for the gender different rates on IGD.

## Background

Internet gaming is widely used as a type of recreation. However, some people develop an internet gaming disorder (IGD) due to their excessive and uncontrolled gaming behaviors. In 2018, the World Health Organization has classified IGD as a mental illness (ICD-11: http://www.who.int/features/qa/gaming-disorder/en/). IGD refers to a mental disorder in which excessive and recurrent use of online games and impairs the physical and psychological functions of the individual [1, 2]. Due to the pervasiveness and harmfulness of IGD, it has become a critical issue to which people should pay close attention to [3, 4].

Decision making refers to the process of evaluating or ranking possible action under uncertainty [5], which is crucial to people’s daily life. Addicts always have a degree of decision-making defects, as do individuals with IGD [6, 7]. The decision-making deficits of IGD individuals was associated with the imbalanced coordination between reward seeking and executive function [8]. The reward system is supposed to be the most critical neurogenic basis for addiction and mainly includes the following brain regions: the limbic system, ventral striatum (VS), insula, amygdala and prefrontal cortex [9, 10]. Executive control refers to the ability of individuals to successfully inhibit improper behavior or thoughts in complex environments [11], of which the anterior cingulate cortex (ACC) and dorsolateral prefrontal cortex (DLPFC) are important components [12, 13]. Addicts often exhibit high reward sensitivity and low loss sensitivity in conjunction with deficits in executive control, which may contribute to high levels of risk behaviors [14]. For example, IGD individuals cannot control gaming behavior despite clearly understanding the negative effects, and choose to indulge in instant pleasure [15]. Therefore, it is very important to study the decision-making process of IGD, which is contribute to understand and intervene IGD behavior.

As one of the paradigms for decision-making research, the card-guessing task was used to studying reward/loss processing of people [16, 17]. In card-guessing task, participants were asked to choose one of the two cards to turn over. The results (win or lose) will be displayed according to the color of the selected card randomly [16]. Using this task, some neuroimaging studies of substance addiction found that addicted subjects showed high reward sensitivity and showed higher activations in the brain regions associated with rewards such as the striatum and the caudate nucleus [18, 19]. Similar features were also observed in IGD subjects, which showed increased activations in the bilateral striatum, thalamus when facing winning outcomes and decreased activation in the ACC, suggesting that individuals with IGD show enhanced reward sensitivity and decreased loss sensitivity than healthy controls [20, 21].

Most of the participants recruited in previous studies were male, and only a few studies focused on gender differences in the potential neurocognitive mechanisms of IGD. Actually, gender is one of the important influencing factors of IGD. The National Institutes of Health (NIH) in the United States encourages the investigation of gender-related differences and hormonal effects in addiction studies [22]. Abundant studies have also revealed that there are significantly more male adolescents with IGD than female adolescents [23–25], which indicated that males are more likely to be addicted to games than females. Some researchers believe that gender differences are quite important in exploring the mechanism of addictive disorders (including substance addiction and behavioral addiction) [26, 27]. A neuroimaging study have reported brain regions implicated in executive control (e.g. DLPFC) and reward processing (e.g. *striatum*, thalamus) showed changes in functional connections (FC) that varied by gender [28]. Another study presented participants with game-related stimuli and asked them to complete a simple cognitive task, the results showed that male players reported stronger craving for game stimuli than female players, accordingly increased brain activations in the striatum and the orbitofrontal cortex (OFC) which are involved in reward processing were observed in male player [29]. However, there is no empirical research to exploring the differences in the neural mechanisms between males and females in decision-making process among IGD groups.

The present study aimed to investigate the differences in neural mechanisms of reward/loss process between males and females with IGD by a card-guessing task. The gender difference was considered an essential dimension in addictive studies [30, 31]. A survey found that male tend to spend more time on games than females [22]. Study have shown that male players seem to be more sensitive to game rewards during the mandatory break [28]. Therefore, our first hypothesis is that male game players are more sensitive to rewards and less sensitive to loss than females in the card-guessing task. Given that previous studies have observed significant gender differences in the whole reward network (including the thalamus and caudate nucleus), which mediates rewards and addiction [32–34], we suspected that increased activation in reward-related brain regions (such as the caudate nucleus and thalamus) could be observed in males when they facing the “win”, and decreased activation in the ACC and DLPFC were observed when they facing the “lose”. In addition, a gambling study showed that the winning outcome of risk decision-making would induce stronger rewards for male gamblers than for female gamblers. That is, male gamblers have higher reward sensitivity [35]. As a kind of behavioral addiction, we also assumed that male IGD subjects induced stronger reward when facing winning results than female subjects in IGD group. Related brain regions (such as the thalamus) will be activated in males with IGD when they face winning outcomes compared to females with IGD. Similarly, females showed more aversion to loss than males at all ages [36]. Males with IGD may showed less sensitive to loss than females and related brain activation in the prefrontal cortex was observed.

## Methods

### Participants

One hundred and eleven right-handed participants were recruited from universities by online advertisement in Shanghai, China, including 54 IGD participants (male: 29; female: 25) and 57 recreational internet game users (RGU) (male: 36; female: 21). RGU refers to those who play internet games but do not show any symptoms of physical or psychological dependence on online games [37]. In the current study, RGU participants can be used as the control group for IGD, which can better explore the neural basis of IGD. All subjects had normal or corrected to normal vision. The final participants were selected through Young’s Internet Addiction Test (IAT) [38] and nine diagnostic criteria for IGD proposed by the DSM-5 Committee [39]. The detailed selection criteria refer to articles by Wang et al. [40]. We determined IGD participants according to the following inclusion criteria: (1) scored higher than 50 on Young’s IAT; (2) met at least 5 DSM-5 criteria; and (3) were familiar with the game League of Legends (Riot Games, Inc.); (4) have played online games more than 14 h per week, for a minimum of 2 years. We selected RGU participants based on the following inclusion criteria: (1) scored lower than 50 on Young’s IAT; (2) met fewer than 5 DSM-5 criteria; and (3) have played online games more than 14 h per week, for a minimum of 2 years. Participants ensured that they did not take any medicine or substances including tea and coffee on the day of scanning. And all participants were instructed not to use any substance of abuse, including caffeinated beverages. No participants reported having previously used illicit drugs (such as cocaine, marijuana) or tobacco. All subjects fit the following criteria: right-handed university students, normal or corrected-to-normal vision, no reported history of illegal drug use, scored lower than 5 on the Beck Depression Inventory questionnaire [41], and no Axis-I psychiatric disorders as per assessment from a 15-min structured psychiatric interview (MINI) [42]. See detailed demographic information in Table 1.

**Table 1:** Demographic information and group differences

	IGD		RGU		***F***	***P***
	Male (***n*** = 29)	Female (***n*** = 25)	Male (***n*** = 36)	Female (***n*** = 21)		
Age (mean ± SD)	22.77 ± 2.22	21.25 ± 1.56	23.33 ± 2.04	22.37 ± 1.88	0.032	0.992
IAT score (mean ± SD)	74.86 ± 12.33	66.84 ± 10.07	31.86 ± 9.31	53.81 ± 11.32	52.175	0.000***
DSM-5 score (mean ± SD)	5.80 ± 1.68	5.72 ± 1.07	2.45 ± 1.61	2.41 ± 1.30	44.989	0.000***
Game playing per week (hours) (mean ± SD)	18.33 ± 2.66	17.50 ± 3.39	18.17 ± 1.83	17.33 ± 2.33	0.197	0.898
Gaming history (months) (mean ± SD)	27.38 ± 10.30	24.95 ± 7.05	25.50 ± 9.01	25.94 ± 8.21	0.259	0.855
Educations (years) (mean ± SD)	14.72 ± 1.41	14.56 ± 1.50	15.22 ± 1.31	14.72 ± 1.32	0.780	0.509

*IGD*, Internet gaming disorder, *RGU* Recreational Internet game users, *IAT* Internet addiction test

∗∗∗*p* < 0.001

### Task and procedure

It takes approximately15 minutes for each participant to complete the entire experiment. The current task refers to the card-guessing task designed by Dong et al. to create winning and losing situations. Participants need to practice a short card-guessing task before the formal experiment, which aims to familiarize the participants with the formal experimental process [20].

The cards used in the present study were the J, Q, K of red hearts, spades, clubs and diamonds, for a total of 12 cards, and the diamonds and red hearts were red cards, while the clubs and spades were black cards. The participants were told to select the card on the left or right by pressing the button (“1” refers to the left card, “2” refers to the right card), one of which was a red card. If the selected card were red, they would win and vice versa.

**Fig. 1:**
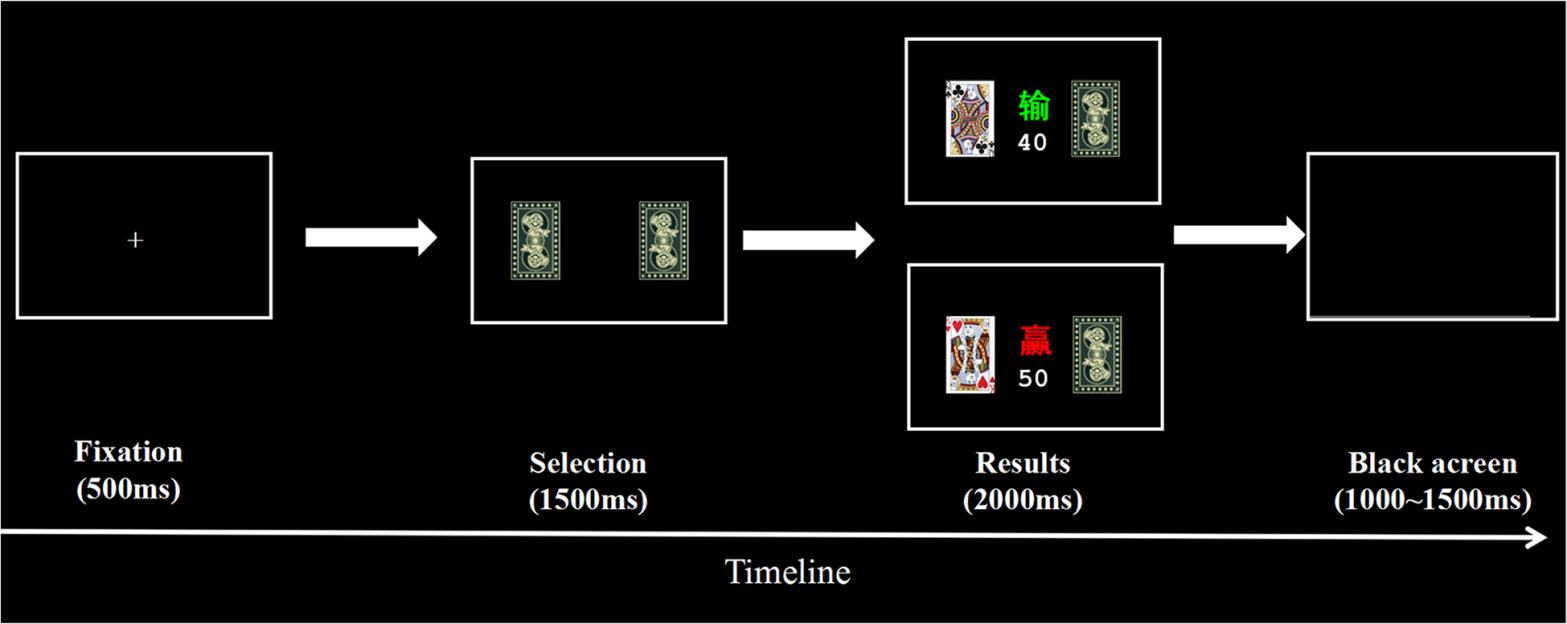
The timeline of one trial in the card-guessing task. First, a fixation was presented for 500 ms. The backs of the two playing cards were then presented for 1500 ms, and the participants were allowed to guess which card was a red card using buttons. Then, the selected card flipped over in the results phase which lasted approximately 2000 ms (“赢” refers to “win”; “输” refers to “loss”). Finally, a black screen of 500 ~ 1000 ms was presented

The specific procedure is shown in Fig. 1. In each trial of the card-guessing task, the fixation is first presented on the screen for 500 ms. Immediately thereafter, the backs of the two playing cards are presented for 1500 ms, and the participants are allowed to guess which card was a red card by the buttons (“1” refers to the left card, “2” refers to the right card). Then, the selected card flips over in the feedback phase which lasted approximately 2000 ms. The feedback presented depends on whether the selected card is a red playing card. If the selected card is red, it represents “win 10 yuan”, and if the selected card is black, it represents “loss 10 yuan”. The feedback presented depends on the color of the card and the participant either wins (selected playing cards are red) 10 yuan or loses (selected playing cards are black) 10 yuan. Next, a black screen is presented for 1000 ~ 1500 ms. The whole task consisted of 144 trials, and the results of loss or win were presented randomly in this experiment.

At the beginning of each study, each participant started with a principal of 50 yuan and was explicitly told that he would receive the full cash balance at the end of the scanning. If the participant misses the button during the selection, the result will also be “loss”. This procedure enabled us to control the sequence of wins and losses fully, and yet the participants think that was their choice. Since the reward is probabilistic, the proportion of positive and negative outcomes experienced by each participant was not exactly 1: 1, there are slight differences between the different participants.

### Image acquisition and preprocessing

Blood oxygen level dependence (BOLD) functional images were acquired using a 3.0- Tesla scanner (Siemens Trio) with gradient echo planar (EPI) T2*-weighted sensitive pulse sequence covering 33 interleaved axial slices of 3 mm thickness with the following parameters: repetition time (TR) = 2000 ms, echo time (TE) = 30 ms, flip angle = 90°, and 64 × 64 matrix with field of view = 220 mm × 220 mm. A high-resolution T1-weighted structural scan was subsequently acquired for each participant. Stimuli were presented using the Invivo synchronous system (Invivo Company, http://www.invivocorp.com/) through a monitor in the head coil.

Preprocessing and statistical analyses of functional MRI data were conducted with SPM8 (https://www.fil.ion.ucl.ac.uk/spm/software/spm8/) and NeuroElf (http://www.neuroelf.net). All functional images were slice-time corrected concerning the first slice acquired, corrected for motion artifacts by realignment to the first volume, and spatially normalized to a standard T1-weighted template with a voxel size of 3 × 3 × 3 mm^3^. Then a 6 mm FWHM Gaussian kernel was used for spatial smoothing. No participants were excluded from further analysis due to the maximum translation that exceeds 2.5 mm or maximum rotation that exceeds 2.5 degrees for further analysis.

### First-level fMRI analysis

Each participant’s data set was then subjected to an event-related analysis. A general linear model (GLM) was applied to assess task-related changes in BOLD signals. Modeled task events included two types: win and loss. However, participants might miss some trials during their selection, and the missed trials were treated as loss in our study. Six head-movement parameters derived from the realignment were included as covariates. All regressors were subsequently convolved with the canonical hemodynamic response function (HRF). A high-pass filter with a cut-off of 128 s was applied to improve the signal-to-noise ratio. Contrast images were calculated based on the parameter estimates output by the general linear model and were passed in a second level group analysis.

### Second-level fMRI analysis

First-level contrasts were submitted to second-level random effects analysis of variance for group analyses. A voxelwise 2 × 2 (group: IGD, RGU; sex: male, female) ANOVA was administered to examine statistically significant between-group differences in the outcome of win. Maps were initially thresholded at *P* < 0.005, and significant voxels were subsequently identified using a joint voxel and extent threshold that corresponded to corrected *P* < 0.05 as determined by the 3dClusterSim (https://afni.nimh.nih.gov/pub/dist/doc/program_help/3dClustSim.html). The cluster extent threshold was 51 voxels (smoothness estimate: 8.4 mm). For the outcome of loss data, processing steps are consistent with the win condition.

### Correlation analyses between behavioral and brain performances

IAT score can indicate the degree of IGD to some extent: the higher the score, the deeper the degree of IGD. In the present task, we respectively compared the different brain activation in the interaction between group and gender during participants facing the win and facing the loss, and took the surviving clusters as ROIs for further analyses. A representative BOLD beta value (signal change) was obtained by averaging the signal of all the voxels within the ROI, and then performing a correlation analysis between the BOLD signal and the IAT score.

## Results

### Brain responses when facing a win

#### Main effects in win conditions

**Fig. 2:**
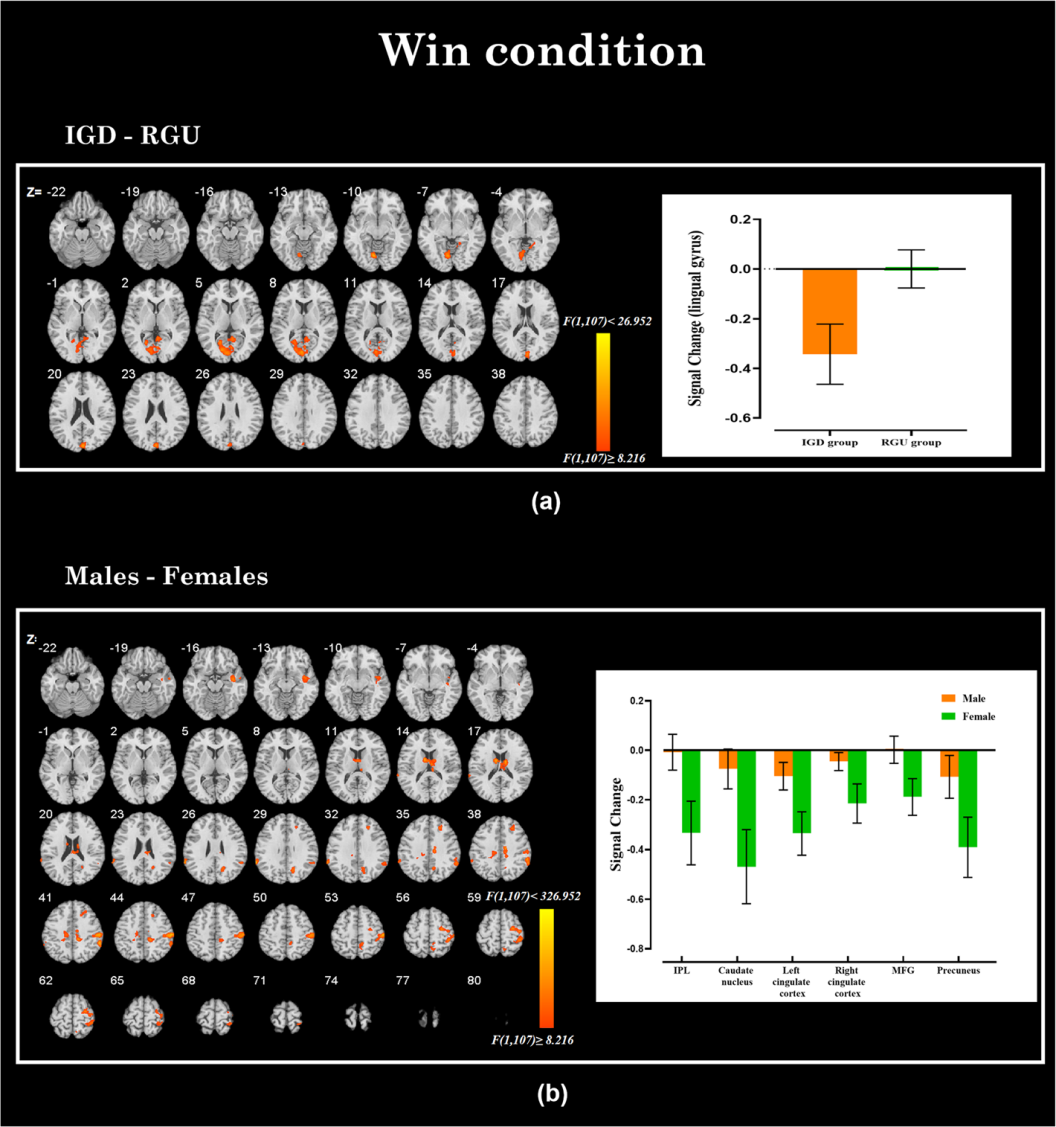
Brain regions showing significant differences by group and gender in winning conditions. **a** The main effect in the group: the IGD subjects showed abnormal activation (shown in yellow) in the left lingual gyrus compared to the RGU group. **b** The main effect in gender: the males showed abnormal activation (shown in yellow) in the left caudate nucleus, bilateral cingulate gyrus, right MFG and right precuneus compared to females

In the win condition, when compared to the RGU group, the IGD group showed a hypoactive BOLD signal in the left lingual gyrus (see Fig. 2a and Table 2).

**Table 2:** Brain regions showing significant group and gender difference in BOLD signal

Condition	Regions	BA^a^	x, y, z^b^	Max ***F***	Number of voxels	Hemisphere^c^
**Win**	**Group**					
	Lingual gyrus	18	-6, 72, −9	19.25	418	L
	**Gender**					
	Caudate nucleus		-9, −3, 15	19.57	125	L
	Cingulate gyrus	24	12, −15, 39	18.1	100	R
	Cingulate gyrus	31	−15, −24, 39	12.47	53	L
	Middle frontal gyrus	9	24, 30, 36	14.23	52	R
	Precuneus	31	15, −66, 30	13.79	51	R
	Inferior parietal lobule	40	60, −24, 51	20.64	223	R
	**Group×Gender**					
	Thalamas		−12, −39, 12	18.94	188	L
	Parahippocampus	30	24, −45, 12	15,99	64	R
	IFG	44	51, 12, 15	15.31	53	R
**Lose**	**Group**					
	Lingual gyrus	18	−9, −72, −9	23.93	447	L
	Parahippocampus	30	−15, −33, −12	16.38	51	L
	ACC	24	6, 36, −3	23.93	82	R
	**Gender**					
	Middle Occipital gyrus	19	42, −78, 6	19.21	203	R
	Caudate nucleus		−3, 9, 12	19.64	113	L
	**Group×Gender**					
	Lingual gyrus		15, −75, −3	17.32	131	R
	IFG	44	51, 12, 15	16.9	51	R

^a^ Brodmann’s area

^b^ Peak Montreal Neurological Institute (MNI) coordinates

^c^ The activation area was on the right side (R) or the left side (L)

We further examined the difference in brain activities between males and females. Males showed hyperactive BOLD signals in the left caudate nucleus, bilateral cingulate gyrus, right middle frontal gyrus (MFG), right precuneus and inferior parietal lobule (IPL) relative to the females (see Fig. 2b and Table 2).

#### Interaction effects in win conditions

**Fig. 3:**
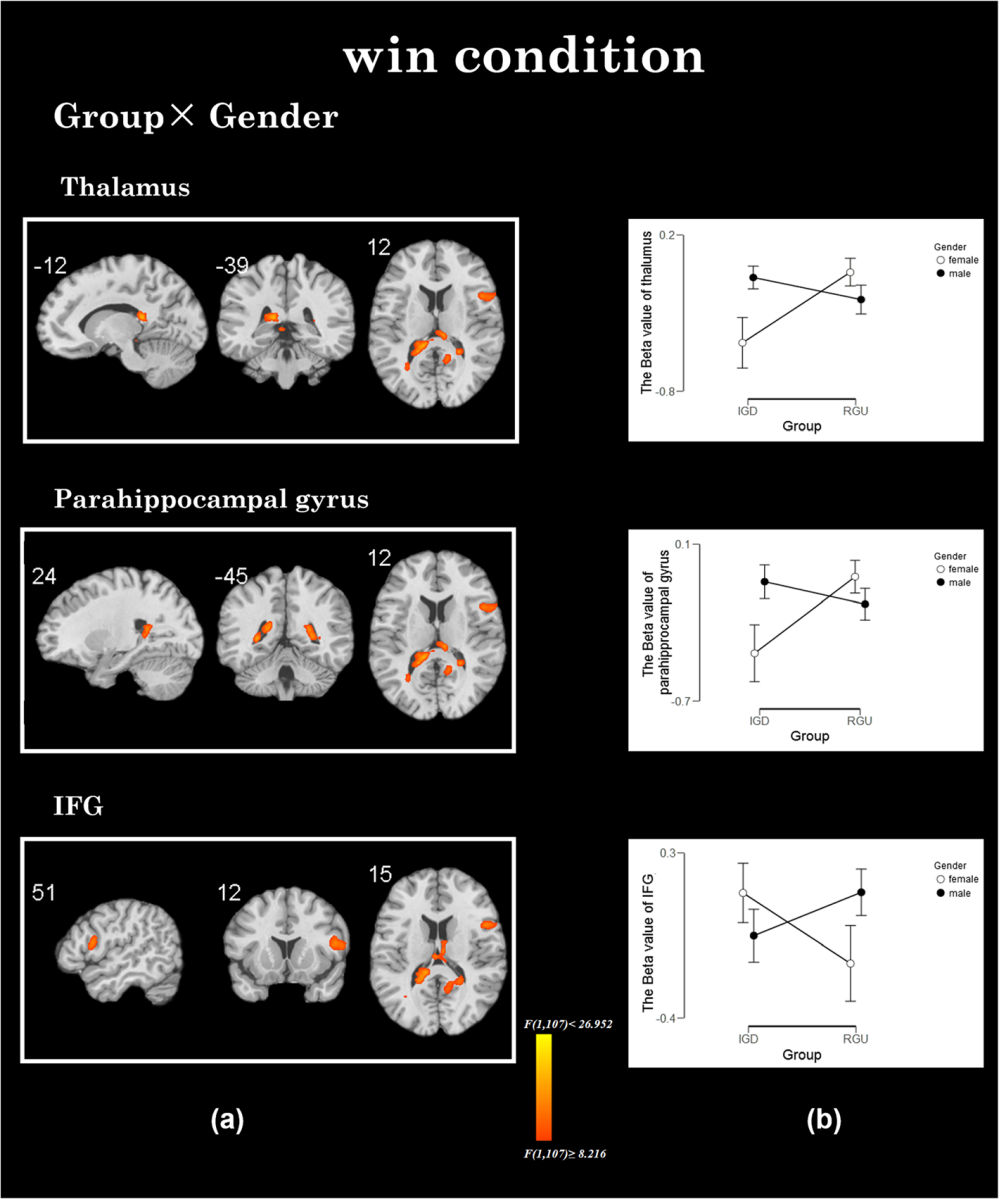
The interaction between group× gender in winning conditions. **a** The interaction effect in group× gender: the brain areas with interactive effects are thalamus, parahippocampal gyrus and IFG. **b** Males with IGD showed increased activation in the thalamus, parahippocampal gyrus and decreased activation in the IFG compared to females with IGD.

Group × gender effects were found in the thalamus, parahippocampus and inferior frontal gyrus (IFG) in win conditions. We extracted these beta values of the thalamus, parahippocampus and IFG for further analysis. Post-hoc test found that IGD participants have lower activation in the thalamus and parahippocampus gyrus (*t = − 2.923, p = 0.004*), males have higher activation in the thalamus and parahippocampus gyrus (*t = 2.293, p = 0.024*). However, there is no significant difference in the IFG activity between different groups (*t = 0.962, p = 0.338*) or different genders (*t = 1.009, p = 0.315*). The simple effect analysis showed that in the IGD group, males showed higher brain activities in the thalamus and lower brain activities in IFG than females. In the RGU group, the results are reversed (see Fig. 3 and Table 2).

### Brain responses when facing a loss

#### Main effects in loss conditions

**Fig. 4:**
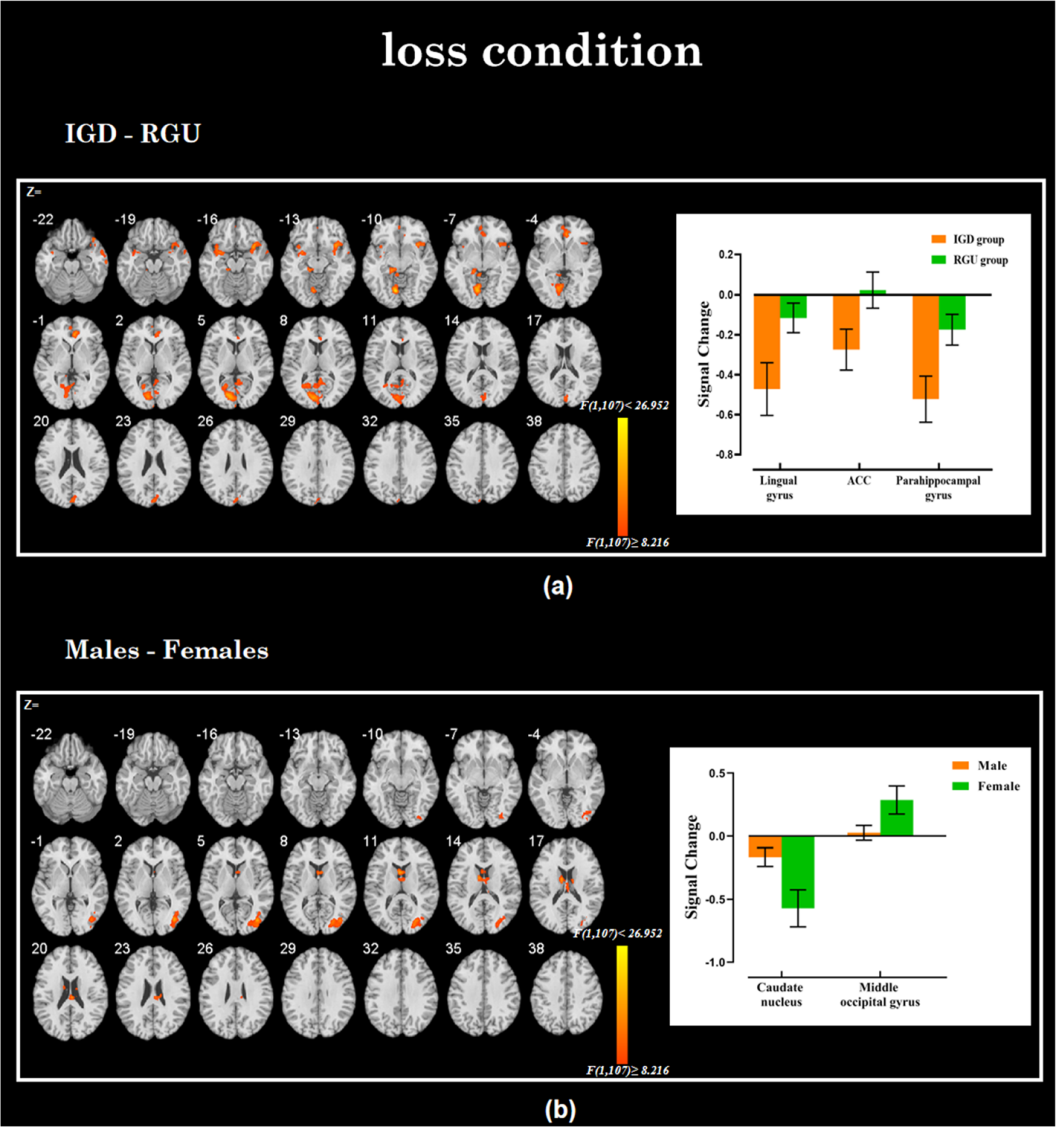
Brain regions showing significant differences by group and gender in losing condition. **a** The main effect by group: the IGD subjects showed abnormal activation (shown in yellow) in the left lingual gyrus, left precuneus and rACC compared to the RGU group. **b** The main effect by gender: the males showed abnormal activation (shown in yellow) in the caudate nucleus and right middle occipital gyrus compared to females

In the loss condition, we first compared the brain activities between the IGD and RGU groups. The IGD group showed hypoactivity in the left lingual gyrus, left parahippocampal gyrus, and right ACC compared to the RGU group (see Fig. 4a and Table 2). Then we examined the difference in brain activities between males and females. Males showed hyperactive left caudate nucleus and decreased BOLD signal activation in the right middle occipital gyrus relative to females (see Fig. 4b and Table 2).

#### Interaction effects in loss conditions

**Fig. 5:**
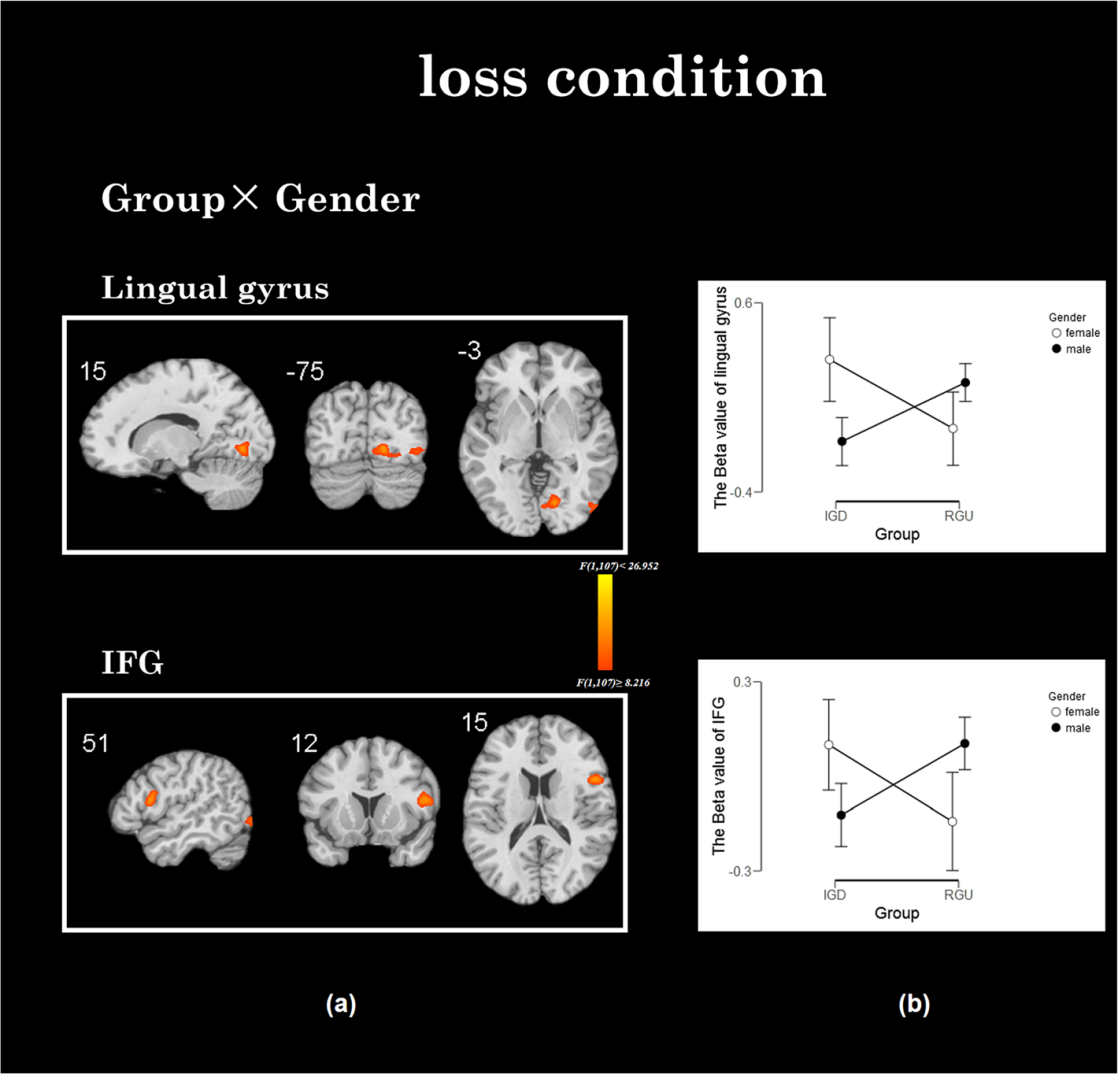
The interaction between group× gender in losing conditions. **a** The interaction effect of group×gender: The brain area with the interactive effect is IFG and lingual gyrus. **b** The males with IGD showed decreased activation in IFG and lingual gyrus comparing to females with IGD

Group and gender have an interaction effect in the lingual gyrus and IFG in the loss condition. Similarly, we extracted these beta values of the lingual gyrus and IFG for further analysis. Post-hoc test found there is no significant brain activations difference in the IFG and lingual gyrus between different groups (lingual gyrus: *t = 0.352, p = 0.725*; IFG: *t = 0.145, p = 0.885*) or different genders (lingual gyrus: *t = − 1.246, p = 0.216*; IFG: *t = 0.216, p = 0.829*). The simple effect analysis showed that compared to females with IGD, males with IGD showed decreased brain activities in the IFG. In the RGU group, the results are reversed (see Fig. 5 and Table 2).

### Correlation results

**Fig. 6:**
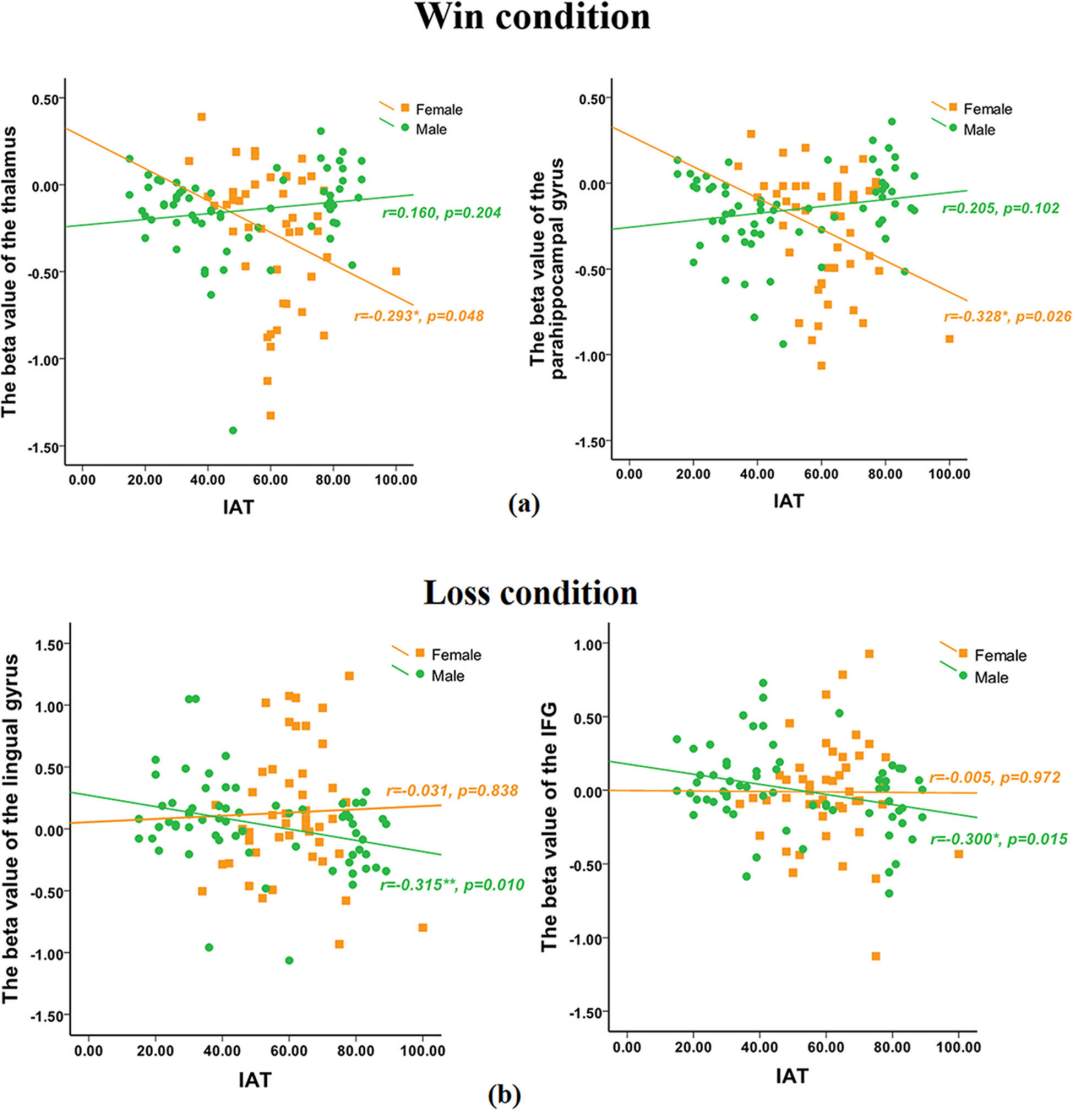
The correlation between IAT and ROIs. **a** When participants facing the win, a significant negative correlation was found between IAT scores and activation of the thalamus, parahippocampal in the female group but not the male group. **b** When they facing the loss, a significant negative correlation was found between IAT scores and activation of the lingual gyrus, IFG in the male group but not female group

When participants facing the win, a significant negative correlation was found between IAT scores and activation of the thalamus (*r = − 0.293, p < 0.05*), parahippocampal (*r = − 0.328, p < 0.05*) in the female group but the not male group (see Fig. 6a). And when they facing the loss, a significant negative correlation was found between IAT scores and activation of the lingual gyrus (*r = − 0.315, p < 0.05*), IFG (*r = − 0.300, p < 0.05*) in the male group but not female group (see Fig. 6b).

## Discussion

In this study, we explore the reward/loss process based on the group and gender for subjectively experienced monetary loss and reward while performing card-guessing tasks. The results showed that: In the face of reward, IGD group and RGU group have no significant difference in reward processing. Male showed increased activation in the reward-related brain regions. And group-by-gender interactions were observed in the thalamus, papahippocampus and IFG. In the face of loss, IGD participants shows decreased activation in the ACC and parahippocampal gyrus than RGUs in loss process, males showed increased activation in the caudate nucleus and decreased activation in the middle occipital gyrus than females, which indicated that IGD group and males do not care about the current loss. At the same time, group-by-sex interaction in the IFG was observed.

### Win condition

In the present study, the hypoactivity in the left lingual gyrus was observed when IGD participants faced a winning outcome. The lingual gyrus has always been reported to be involved in visual recognition and is believed to play a role in episodic memory consolidation [43, 44]. The hypoactivity in the lingual gyrus indicated that IGD participants were not sensitive to the visual stimuli presented by the screen, which may be related to their long-term viewing of computer screens. The lingual gyrus is involved in basic visual processing, so although there were many addictive studies showed that addicts usually have abnormal activation in lingual gyrus [43, 45–47]. However, no research have explored it further. Inconsistent with prior studies [48–50], there appeared to be no difference in reward processing between the two groups. The possible cause was that we used RGU participants as a control group in the current study. RGU participants are also big enthusiasts of online games, similar to those with IGD. Thus, there is no group difference in reward processing.

Then, we compared the differences in brain responses between males and females when rewards were met in the card-guessing task. Males showed a hyperactivity in the caudate nucleus, cingulate gyrus, MFG, precuneus and IPL than females. The caudate nucleus is a crucial brain area for dopamine delivery and is used to strengthen reward expectations [51]. In reward processing, the received reward information is finally transmitted to prefrontal cortex and cingulate cortex for further evaluation and processing [52]. The MFG is more sensitive to social benefits, and cingulate gyrus plays an important role in value evaluation [53–55]. In this study, the increased activation in these reward-related brain regions in male players indicated that their motivation and impulse to pursue rewards are enhanced [56–58]. The precuneus is believed to be associated with pleasure, and studies have found that risk decisions are usually accompanied by high activation of the IPL and precuneus [59, 60]. Therefore, we speculate that males players may prefer risk decision-making activities. Anyway, these results suggested that strong rewards and the impulse to pursue rewards [61] seem to be more pronounced in males. This may be the reason why male players are more likely to have IGD.

In addition, consistent with our hypothesis, the present study found that gender (male, female) and group (IGD, RGU) have significant interactions in the thalamus, parahippocampal gyrus and IFG in the win condition. Further comparisons showed increased brain activities in the thalamus and parahippocampal gyrus in males with IGD as compared to females with IGD. The thalamus plays an important role in reward processing [62, 63] and goal-directed behaviors, alongside many other cognitive and motor functions [64]. The thalamus is a part of the cortico-striato-thalamo-cortical circuits underlying both reward and motivated behaviors [58]. The parahippocampus gyrus participates in the memory of rewards [65, 66]. A study found that injecting maca into the parahippocampus can cause CPP and self-injection behavior, indicating that the hippocampus participates in reward processing [67]. Abnormal activities of the parahippocampus gyrus and thalamus in drug addict indicate that they easily perceive and seek rewards [68]. Hence the neural activities in the face of winning results reflect that males with IGD seem to be able to experience a higher sense of reward that drives them to seek rewards. Additionally, lower IFG activity was observed in males with IGD. IFG is mainly involved in the executive control processing and reward/punishment information evaluation, the execution control tasks often weaken the function and activation of IFG, which leads to failure of self-control [69, 70]. The fMRI study also found that the degree of IFG activation significantly predicted the self-control behavior of individuals in real life [71, 72]. In the present study, males with IGD showed weakened activities in the IFG indicating that they showed a lower sense of control over the winning outcomes (rewards) compared to females with IGD. Males with IGD indulge in the pleasure brought by reward and difficult to control. In a word, the motivation to obtain rewards represents a central feature of addictions and seems to be more pronounced in males with IGD. At the same time, they cannot suppress and deal with the motivation of pursuing reward well. This may be the reason why the number of males with IGD is higher - males with IGD more difficult to quit the game than females.

However, the present results showed that male and female brain activations in the RGU group were opposite to those in the IGD group, which is different from other studies. Compared with the males, female RGUs seem to be more sensitive to winning. We cannot provide a reasonable explanation based on the current data. The specific reasons need to be further explored.

### Loss condition

In the loss condition, we observed hypoactivity in the left lingual gyrus, right ACC and left parahippocampal gyrus in the IGD group than the RGU group. Unlike the results of facing win, IGD has more hypoactive reward-related brain regions including the rACC and parahippocampal gyrus in the loss condition which means that IGD has lower loss sensitivity than RGU participants. The ACC is considered to be a part of an integral network involved in decision-making and evaluation, but there is still much debate about its role [73]. Early card guessing task found that the dorsal ACC activity was associated with greater “uncertainty” of outcome [74]. The ACC plays an essential role in the motivation, cognition and action [75] and also in executive control and reward evaluation [21]. Several neuroimaging studies have examined the impaired risk assessment capabilities by dysfunction or structural abnormalities in ACC in people with IGD [76, 77]. The more hypoactive ACC were observed in the IGD indicated that they could not make an accurate assessment of the risk outcome or were not adequately sensitive to loss.

We also found that males showed hyperactivity in the left caudate nucleus and hypoactivity in the right middle occipital gyrus relative to the females. The right middle occipital gyrus is related to visual information processing and visual space attention [78]. Males showed a hypoactive right middle occipital gyrus might suggesting that females have better visual information processing ability relative to males. That may be due to innate gender differences. Similar to the response to winning, the hyperactivity in the caudate nucleus that involved in reward process was also observed in males when they facing the loss, which indicated that males do not pay much attention to the current loss, even the current loss have induced their reward expectations for the next card-guessing task.

There also has an interaction in the IFG and lingual gyrus in the loss condition. We also found decreased activities in the IFG in males with IGD in the loss condition compared to females with IGD. The IFG was associated with executive control, when the IFG was involved in the punishment process, the individual can be controlled not to take risks in order to avoid loss [79, 80]. Therefore, the lower activation in the IFG in males with IGD indicated that their ability of evaluate loss is insufficient, that is, males with IGD are insensitive to the losses relative to the females. In the RGU group, the results also reverse to the IGD group. These results indicated that females with RGU appear to exhibit higher reward sensitivity and insensitivity to loss than males, which is inconsistent with our expectation and previous research. We cannot provide a reasonable explanation based on the current data. We need to explore the reason in further research.

Following previous research, the present study proved that reward/loss processing is always associated with abnormal activation in the limbic system (thalamus, ACC, parahippocampal gyrus) and prefrontal cortex. The above results indicate that males with IGD were induced with a higher reward when they saw the winning outcome. Furthermore, the loss insensitive feature in males with IGD was observed when they were facing the loss [81]. We suspect that this may be the reason why the males with IGD find it easy to indulge in the game and more difficult to withdraw from the game.

### Different gender showed opposite activation patterns in IGD degree and brain region response

A significant negative correlation was found between IAT scores and activation of the thalamus, parahippocampal in the female group but not male group when participants facing the win. This result showed the higher the degree of IGD in females, the lower the activation in the thalamus and parahippocampus gyrus. This means that with the increase of IGD level in the female group, they seem to be less and less sensitive to rewards. However, in the male group, although the degree of IGD did not reach a significant correlation with the activities of the thalamus and parahippocampus gyrus, it showed a positive trend. In addition, a significant negative correlation was found between IAT scores and activation of the lingual gyrus, IFG in the male group but not female group when they facing the loss. That showed the higher the degree of IGD in males, the lower the activation in the IFG and indicated that with the increase of IGD level in the male group, they seem to be less and less sensitive to losses. Therefore, we infer that different gender showed opposite activation patterns in IGD degree and rewards/losses processing. But we cannot provide a reasonable explanation based on the current data, the specific reasons need to be further explored.

### Limitations

The current study has several limitations. First, behavioral performance during the game was not collected; therefore, we cannot relate neural discovery to game performance. Second, there appeared to be no difference in reward processing between the two groups. We speculated that the possible cause was that we used RGU participants as a control group and we will add a healthy control group in the following study to further explore the reasons. Third, due to the limitation of experimental procedures, control condition cannot be set, but we expect to balance the differences with large samples of participants. Finally, the present results showed that male and female brain activations in the RGU group were opposite to those in the IGD group, which is different from other studies and our expectation. At present, we cannot provide a reasonable explanation based on current data and we believe a further study focusing on the female IGD and female RGU could provide more evidence for an explanation. Additionally, we plan to recruit more female subjects to explore this issue.

## Conclusions

In conclusion, there appeared to be no difference in reward processing between the IGD and RGU group, but IGD showed less sensitivity to loss. And male players showed more sensitivity to rewards and less sensitivity to losses. Last but not least, males and females showed opposite activation patterns in IGD degree and rewards/losses processing. And male IGD subjects are more sensitive to reward and less sensitive to loss than females, which might be the reason for the gender different rates on IGD.

## Data Availability

The datasets used and/or analyzed during the current study are available from the corresponding author on reasonable request.

## Abbreviations

IGD: Internet gaming disorder
RGU: Recreational internet game user
VS: Ventral striatum
ACC: Anterior cingulate cortex
DLPFC: Dorsolateral prefrontal cortex
NIH: National Institutes of Health
OFC: Orbitofrontal cortex
IAT: Internet Addiction Test
BOLD: Blood oxygen level dependence
GLM: General linear model
fMRI: Functional Magnetic Resonance Imaging
HRF: Hemodynamic response function
MFG: Middle frontal gyrus
IFG: Inferior frontal gyrus
IPL: Inferior parietal lobule
FC: Functional connections

## References

[1] King DL, Delfabbro PH, Potenza MN, Demetrovics Z, Billieux J, Brand M (2018). Internet gaming disorder should qualify as a mental disorder. Aust N Z J Psychiatry.

[2] Anderson EL, Steen E, Stavropoulos V (2017). Internet use and problematic internet use: a systematic review of longitudinal research trends in adolescence and emergent adulthood. Int J Adolesc Youth.

[3] Faust KA, Prochaska JJ (2018). Internet gaming disorder: a sign of the times, or time for our attention?. Addict Behav.

[4] Greenfield DN (2018). Treatment considerations in internet and video game addiction: a qualitative discussion. Child Adolesc Psychiatr Clin N Am.

[5] Malakooti B (2012). Decision making process: typology, intelligence, and optimization. J Intell Manuf.

[6] Li Y, Ramoz N, Derrington E, Dreher J-C. Hormonal responses in gambling versus alcohol abuse: a review of human studies. Prog Neuro-Psychopharmacol Biol Psychiatry. 2020;100. 10.1016/j.pnpbp.2020.109880. 10.1016/j.pnpbp.2020.109880 32004637

[7] Balogh KN, Mayes LC, Potenza MN (2013). Risk-taking and decision-making in youth: relationships to addiction vulnerability. J Behav Addict.

[8] Brand M, Wegmann E, Stark R, Mueller A, Woelfling K, Robbins TW (2019). The interaction of person-affect-cognition-execution (I-PACE) model for addictive behaviors: update, generalization to addictive behaviors beyond internet-use disorders, and specification of the process character of addictive behaviors. Neurosci Biobehav Rev.

[9] Chiew KS, Braver TS (2011). Positive affect versus reward: emotional and motivational influences on cognitive control. Front Psychol.

[10] Romer AL, Kang MS, Nikolova YS, Gearhardt AN, Hariri AR (2019). Dopamine genetic risk is related to food addiction and body mass through reduced reward-related ventral striatum activity. Appetite.

[11] Zhang J, Hu Y, Li H, Zheng H, Xiang M, Wang Z (2020). Altered brain activities associated with cue reactivity during forced break in subjects with internet gaming disorder. Addict Behav.

[12] Dong G-H, Wang M, Wang Z, Zheng H, Du X, Potenza MN. Addiction severity modulates the precuneus involvement in internet gaming disorder: functionality, morphology and effective connectivity. Prog Neuro-Psychopharmacol Biol Psychiatry. 2020;98. 10.1016/j.pnpbp.2019.109829. 10.1016/j.pnpbp.2019.109829 31790725

[13] Dong G-H, Wang M, Zheng H, Wang Z, Du X, Potenza MN. Disrupted prefrontal regulation of striatum-related craving in internet gaming disorder revealed by dynamic causal modeling: results from a cue-reactivity task. Psychol Med. 2020:1–13. 10.1017/s003329172000032x. 10.1017/S003329172000032X 32102722

[14] Kahn RE, Chiu PH, Deater-Deckard K, Hochgraf AK, King-Casas B, Kim-Spoon J (2018). The interaction between punishment sensitivity and effortful control for emerging adults’ substance use behaviors. Subst Use Misuse.

[15] Yau Y, Potenza M (2014). Internet gaming disorder. Psychiatr Ann.

[16] Reuter J, Raedler T, Rose M, Hand I, Glascher J, Buchel C (2005). Pathological gambling is linked to reduced activation of the mesolimbic reward system. Nat Neurosci.

[17] Van de Steen F, Krebs RM, Colenbier N, Almgren H, Marinazzo D. Effective connectivity modulations related to win and loss outcomes. Neuroimage. 2020;207. 10.1016/j.neuroimage.2019.116369. 10.1016/j.neuroimage.2019.116369 31747561

[18] Crane NA, Gorka SM, Weafer J, Langenecker SA, de Wit H, Phan KL (2018). Neural activation to monetary reward is associated with amphetamine reward sensitivity. Neuropsychopharmacology.

[19] Lessov-Schlaggar CN, Lepore RL, Kristjansson SD, Schlaggar BL, Barnes KA, Petersen SE (2013). Functional neuroimaging study in identical twin pairs discordant for regular cigarette smoking. Addict Biol.

[20] Dong G, Li H, Wang L, Potenza MN. Cognitive control and reward/loss processing in Internet gaming disorder: results from a comparison with recreational Internet game-users. Eur Psychiatry. 2017;44:30–8. 10.1016/j.eurpsy.2017.03.004 28545006

[21] Dong G, Huang J, Du X (2011). Enhanced reward sensitivity and decreased loss sensitivity in internet addicts: an fMRI study during a guessing task. J Psychiatr Res.

[22] Clayton JA, Collins FS (2014). Policy: NIH to balance sex in cell and animal studies. Nature..

[23] Dong G, Zheng H, Liu X, Wang Y, Du X, Potenza MN (2018). Gender-related differences in cue-elicited cravings in internet gaming disorder: the effects of deprivation. J Behav Addict.

[24] Ha YM, Hwang WJ (2014). Gender differences in internet addiction associated with psychological health indicators among adolescents using a national web-based survey. Int J Ment Health Addict.

[25] Lee S-Y, Lee D, Nam CR, Kim DY, Park S, Kwon J-G (2018). Distinct patterns of internet and smartphone-related problems among adolescents by gender: latent class analysis. J Behav Addict.

[26] Sanchis-Segura C, Becker JB (2016). Why we should consider sex (and study sex differences) in addiction research. Addict Biol.

[27] Tuchman E (2010). Women and addiction: the importance of gender issues in substance abuse research. J Addict Dis.

[28] Dong G, Wang Z, Wang Y, Du X, Potenza MN (2019). Gender-related functional connectivity and craving during gaming and immediate abstinence during a mandatory break: implications for development and progression of internet gaming disorder. Prog Neuro-Psychopharmacol Biol Psychiatry.

[29] Dong G, Wang L, Du X, Potenza MN (2018). Gender-related differences in neural responses to gaming cues before and after gaming: implications for gender-specific vulnerabilities to internet gaming disorder. Soc Cogn Affect Neurosci.

[30] Blanco C, Hasin DS, Petry N, Stinson FS, Grant BF (2006). Sex differences in subclinical and DSM-IV pathological gambling: results from the national epidemiologic survey on alcohol and related conditions. Psychol Med.

[31] Petit G, Luminet O, Uva MCS, Monhonval P, Leclercq S, Spilliaert Q (2017). Gender differences in affects and craving in alcohol-dependence: a study during alcohol detoxification. Alcoholism.

[32] Becker JB, Chartoff E (2019). Sex differences in neural mechanisms mediating reward and addiction. Neuropsychopharmacology.

[33] Becker JB, Perry AN, Westenbroek C. Sex differences in the neural mechanisms mediating addiction: a new synthesis and hypothesis. Biol Sex Differ. 2012;3. 10.1186/2042-6410-3-14. 10.1186/2042-6410-3-14 PMC3724495 22676718

[34] Sawyer KS, Oscar-Berrnan M, Barthelemy OJ, Papadimitriou GM, Harris GJ, Malais N (2017). Gender dimorphism of brain reward system volumes in alcoholism. Psychiatry Res.

[35] Teeters JB, Ginley MK, Whelan JP, Meyers AW, Pearlson GD (2015). The moderating effect of gender on the relation between expectancies and gambling frequency among college students. J Gambl Stud.

[36] Grose-Fifer J, Migliaccio R, Zottoli TM (2014). Feedback processing in adolescence: an event-related potential study of age and gender differences. Dev Neurosci.

[37] Wang Y, Wu L, Wang L, Zhang Y, Du X, Dong G (2017). Impaired decision-making and impulse control in internet gaming addicts: evidence from the comparison with recreational internet game users. Addict Biol.

[38] Young KS (1998). Internet addiction: the emergence of a new clinical disorder. CyberPsychol Behav.

[39] Petry NM, Rehbein F, Gentile DA, Lemmens JS, Rumpf HJ, Mößle T (2014). An international consensus for assessing internet gaming disorder using the new DSM-5 approach. Addiction.

[40] Wang L, Wu L, Wang Y, Li H, Liu X, Du X (2017). Altered brain activities associated with craving and cue reactivity in people with internet gaming disorder: evidence from the comparison with recreational internet game users. Front Psychol.

[41] Beck AT, Ward CH, Mendelson M, Mock J, Erbaugh J (1961). Beck depression inventory (BDI). Arch Gen Psychiatry.

[42] Lecrubier Y, Sheehan DV, Weiller E, Amorim P, Bonora I, Sheehan KH (1997). The Mini international neuropsychiatric interview (MINI): a short diagnostic structured interview: reliability and validity according to the CIDI. Eur Psychiatry.

[43] Kukolja J, Göreci DY, Onur ÖA, Riedl V, Fink GR (2016). Resting-state fMRI evidence for early episodic memory consolidation: effects of age. Neurobiol Aging.

[44] Tao H, Guo S, Ge T, Kendrick KM, Xue Z, Liu Z (2013). Depression uncouples brain hate circuit. Mol Psychiatry.

[45] Han DH, Kim SM, Bae S, Renshaw PF, Anderson JS (2017). Brain connectivity and psychiatric comorbidity in adolescents with internet gaming disorder. Addict Biol.

[46] Starcke K, Antons S, Trotzke P, Brand M (2018). Cue-reactivity in behavioral addictions: a meta-analysis and methodological considerations. J Behav Addict.

[47] Lee D, Namkoong K, Lee J, Jung Y-C (2019). Preliminary evidence of altered gray matter volume in subjects with internet gaming disorder: associations with history of childhood attention-deficit/hyperactivity disorder symptoms. Brain Imaging Behav.

[48] Myers CE, Sheynin J, Balsdon T, Luzardo A, Beck KD, Hogarth L (2016). Probabilistic reward- and punishment-based learning in opioid addiction: experimental and computational data. Behav Brain Res.

[49] Myers CE, Rego J, Haber P, Morley K, Beck KD, Hogarth L (2017). Learning and generalization from reward and punishment in opioid addiction. Behav Brain Res.

[50] Dong G, Wu L, Wang Z, Wang Y, Du X, Potenza MN (2018). Diffusion-weighted MRI measures suggest increased white-matter integrity in internet gaming disorder: evidence from the comparison with recreational internet game users. Addict Behav.

[51] Volkow ND, Wang GJ, Fowler JS, Tomasi D, Telang F (2011). Addiction: beyond dopamine reward circuitry. Proc Natl Acad Sci U S A.

[52] Bermejo PE, Dorado R, Zea-Sevilla MA, Sanchez Menendez V (2011). Neuroanatomy of financial decisions. Neurologia.

[53] Porter BS, Hillman KL, Bilkey DK (2019). Anterior cingulate cortex encoding of effortful behavior. J Neurophysiol.

[54] Shenhav A, Karmarkar UR. Dissociable components of the reward circuit are involved in appraisal versus choice. Sci Rep. 2019;9. 10.1038/s41598-019-38927-7. 10.1038/s41598-019-38927-7 PMC6374444 30760824

[55] Umemoto A, Inzlicht M, Holroyd CB (2019). Electrophysiological indices of anterior cingulate cortex function reveal changing levels of cognitive effort and reward valuation that sustain task performance. Neuropsychologia.

[56] Fumagalli M, Rosa M, Giannicola G, Marceglia S, Lucchiari C, Servello D (2015). Subthalamic involvement in monetary reward and its dysfunction in parkinsonian gamblers. J Neurol Neurosurg Psychiatry.

[57] Fumiko H, Watson CL, Kesler SR, Bettinger KE, Reiss AL (2008). Gender differences in the mesocorticolimbic system during computer game-play. J Psychiatr Res.

[58] Haber SN, Knutson B (2010). The reward circuit: linking primate anatomy and human imaging. Neuropsychopharmacology.

[59] Harrington DL, Boyd LA, Mayer AR, Sheltraw DM, Lee RR, Huang M (2004). Neural representation of interval encoding and decision making. Brain Res Cogn Brain Res.

[60] Sacre P, Kerr MSD, Subramanian S, Kahn K, Gonzalez-Martinez J, Johnson MA (2016). The precuneus may encode irrationality in human gambling. Conf Proc IEEE Eng Med Biol Soc.

[61] Rosell-Negre P, Bustamante JC, Fuentes-Claramonte P, Costumero V, Llopis-Llacer JJ, Barrós-Loscertales A (2016). Reward contingencies improve goal-directed behavior by enhancing posterior brain attentional regions and increasing corticostriatal connectivity in cocaine addicts. PLoS One.

[62] Corbit LH, Muir JL, Balleine BW (2003). Lesions of mediodorsal thalamus and anterior thalamic nuclei produce dissociable effects on instrumental conditioning in rats. Eur J Neurosci.

[63] Huang AS, Mitchell JA, Haber SN, Alia-Klein N, Goldstein RZ. The thalamus in drug addiction: from rodents to humans. Philos Trans R Soc B Biol Sci. 2018;373(1742). 10.1098/rstb.2017.0028. 10.1098/rstb.2017.0028 PMC5790826 29352027

[64] Corbit LH, Muir JL, Balleine BW (2015). Lesions of mediodorsal thalamus and anterior thalamic nuclei produce dissociable effects on instrumental conditioning in rats. Eur J Neurosci.

[65] Dillon DG, Dobbins IG, Pizzagalli DA (2014). Weak reward source memory in depression reflects blunted activation of VTA/SN and parahippocampus. Soc Cogn Affect Neurosci.

[66] Qi X, Yang Y, Dai S, Gao P, Du X, Zhang Y (2016). Effects of outcome on the covariance between risk level and brain activity in adolescents with internet gaming disorder. Neuroimage Clin.

[67] Zarrindast M-R, Nouri M, Ahmadi S (2007). Cannabinoid CB1 receptors of the dorsal hippocampus are important for induction of conditioned place preference (CPP) but do not change morphine CPP. Brain Res.

[68] Mira B, Sabine VDK, Andrea K, Henning B, Reed LJ, Braus DF (2010). Nicotine dependence is characterized by disordered reward processing in a network driving motivation. Biol Psychiatry.

[69] Friese M, Binder J, Luechinger R, Boesiger P, Rasch B (2013). Suppressing emotions impairs subsequent stroop performance and reduces prefrontal brain activation. PLoS One.

[70] Lee N, Chatzisarantis N, Hagger MS (2016). Adequacy of the sequential-task paradigm in evoking ego-depletion and how to improve detection of ego-depleting phenomena. Front Psychol.

[71] Lopez RB, Hofmann W, Wagner DD, Kelley WM, Heatherton TF (2014). Neural predictors of giving in to temptation in daily life. Psychol Sci.

[72] Lopez RB, Milyavskaya M, Hofmann W, Heatherton TF (2016). Motivational and neural correlates of self-control of eating: a combined neuroimaging and experience sampling study in dieting female college students. Appetite.

[73] Ebitz RB, Hayden BY (2016). Dorsal anterior cingulate: a Rorschach test for cognitive neuroscience. Nat Neurosci.

[74] Critchley HD, Mathias CJ, Dolan RJ (2001). Neural activity in the human brain relating to uncertainty and arousal during anticipation. Neuron.

[75] Kennerley SW, Walton ME, Behrens TE, Buckley MJ, Rushworth MF (2006). Optimal decision making and the anterior cingulate cortex. Nat Neurosci.

[76] Dong G, Wang L, Du X, Potenza MN (2017). Gaming increases craving to gaming-related stimuli in individuals with internet gaming disorder. Biol Psychiatry Cogn Neurosci Neuroimaging.

[77] Yan Z (2011). Gray matter abnormalities in internet addiction: a voxel-based morphometry study. Eur J Radiol.

[78] Muller NG, Kleinschmidt A (2003). Dynamic interaction of object- and space-based attention in retinotopic visual areas. J Neurosci.

[79] Hampshire A, Chamberlain SR, Monti MM, Duncan J, Owen AM (2010). The role of the right inferior frontal gyrus: inhibition and attentional control. Neuroimage.

[80] Knutson B, Taylor J, Kaufman M, Peterson R, Glover G (2005). Distributed neural representation of expected value. J Neurosci.

[81] Dong G, Lin X, Zhou H, Du X (2014). Decision-making after continuous wins or losses in a randomized guessing task: implications for how the prior selection results affect subsequent decision-making. Behav Brain Funct.

